# A dual-amplification strategy-intergated SERS biosensor for ultrasensitive hepatocellular carcinoma-related telomerase activity detection

**DOI:** 10.3389/fbioe.2022.1124441

**Published:** 2023-01-13

**Authors:** Kang Shen, Weiwei Hua, Shengjie Ge, Yu Mao, Yuexing Gu, Gaoyang Chen, Youwei Wang

**Affiliations:** ^1^ Department of Neurosurgery, The Affiliated Hospital of Yangzhou University, Yangzhou, China; ^2^ Institute of Translational Medicine, Medical College, Yangzhou University, Yangzhou, China; ^3^ Department of Otorhinolaryngology Head and Neck Surgery, The Affiliated Hospital of Yangzhou University, Yangzhou University, Yangzhou, China; ^4^ Department of Oncology, Taizhou Second People's Hospital, Taizhou, China

**Keywords:** strand displacement amplification, catalytic hairpin assembly, surface-enhanced Raman scattering, telomerase, hepatocellular carcinoma

## Abstract

Telomerase has been considered as a biomarker for early diagnosis and prognosis assessment of hepatocellular carcinoma (HCC), while the highly sensitive and specific methods remain challenging. To detect telomerase, a novel surface-enhanced Raman scattering (SERS) biosensor was constructed using the dual DNA-catalyzed amplification strategy composed of strand displacement amplification (SDA) and catalytic hairpin assembly (CHA). This strategy relies on the extension reaction of telomerase primer induced by telomerase, forming long-stranded DNAs with repetitive sequence to catalyze the follow-up SDA event. Subsequently, the SDA products can trigger the CHA reaction between the SERS probes (Au-Ag nanocages (Au-AgNCs) modified with hairpin DNA1 and Raman reporters) and capture substrate (Au@SiO_2_ array labeled with hairpin DNA2), resulting in the formation of numerous “hot spots” to significantly enhance the SERS signal. Results are promising that the established biosensor presented excellent reproducibility, specificity and sensitivity. Moreover, ELISA was applied as the golden standard to verify the application of the proposed biosensor in real samples and the results confirmed the satisfactory accuracy of our method. Therefore, the proposed SERS biosensor has the potential to be an ideal tool for the early screening of HCC.

## 1 Introduction

Hepatocellular carcinoma (HCC) is the commonest malignancy of the liver with high mortality, whose main risk factors are hepatitis and cirrhosis (Salomao et al., 2012; Midorikawa et al., 2016). Early-stage HCC patients receiving surgical resection or liver transplantation present a relatively high five-year survival rate of 70%, while advanced-stage patients have a poor prognosis due to the lack of early cancer intervention (Roelli et al., 2016; Abara et al., 2020). In this sense, development of relevant biomarkers to assist the early-stage diagnosis is a priority. Telomerase is a ribonucleoprotein enzyme elongating telomeric DNA, whose differential expression in normal tissues and tumor cells makes it an attractive biomarker for HCC diagnostic and prognostic (Wang et al., 2018; Afshari et al., 2022; Manganelli et al., 2022). Researchers have found that quantitative assay of telomerase can provide a satisfactory positive rate for HCC diagnosis compared to alpha-fetoprotein (AFP) and PIVKA-II (Tzartzeva and Singal., 2018). However, current determination of telomerase mainly depends on instrumental detection such as polymerase chain reaction (PCR) based telomere repeat amplification protocol (TRAP), enzyme-linked immunosorbent assay (ELISA) and enhanced chemiluminescence (ECL), which are costly, complicated and insensitive, resulting in the limited clinical application (Ferlicot et al., 1999; Itoi et al., 1999; Huang et al., 2013). consequently, a low-cost, simple, sensitive and specific clinical assay for telomerase is urgently needed.

Surface-enhanced Raman scattering (SERS), a potent technique for detecting trace molecular, has applications in environmental monitoring, chemical and biomolecule analysis, due to its “fingerprinting” capacity to generate distinctive spectra for identifying various compounds (Hu and Zhang., 2010; Chen et al., 2019; Sultangaziyev et al., 2022). The enhancement of SERS mainly originates from highly concentrated electromagnetic mechanism (EM) caused by plasmon excitation in metal nanostructures surface and the composition, structure, and distribution of nanomaterials are the key determinants of EM “hot spots” (Tao and Yang, 2005; Yoshida et al., 2009). Thus, by synthesizing novel nanomaterials with special morphology and porous structure, significant SERS signal enhancement effect can be achieved. As a new type of nano-array, Au@SiO_2_ array consisting of single-layer SiO_2_ microsphere and Au nanoparticles (AuNPs) comes to our attention (Xu et al., 2016; Yang et al., 2016). Due to its rough surface, the local electromagnetic field can be significantly amplified, serving as the “hot spots” for SERS signal enhancement. Moreover, its large specific surface area can provide abundant binding sites for the immobilization of biomolecules. Therefore, Au@SiO_2_ arrays are potential candidates for SERS detection. Au-Ag nanocages (Au-AgNCs) are hollow, porous materials, whose eight vertices and superimposed plasmonic excitonic resonance of inner and outer surfaces can excite the EM, which is generally considered to be the major mechanism for enhancing SERS signaling. Additionally, Au-AgNCs can aggregate to generate a stronger electromagnetic field coupling than individual nanoparticles (Rycenga et al., 2009; Cao et al., 2020). Therefore, it is ideal to prepare SERS probes using Au-AgNCs.

In the last few years, SERS-based detection of telomerase activity has received widespread attention due to its dramatic enhancement effect. However, telomerase is expressed at low levels in early-stage HCC patients and has a complex composition, which still does not meet the requirements for ultra-sensitive detection (Zong et al., 2013; Wang et al., 2021). Therefore, researchers attempt to introduce DNA signal amplification strategy into the SERS-based detection, and strand displacement amplification (SDA) and catalytic hairpin assembly (CHA) stand out from the crowd with the advantages of high sensitivity, specificity and easy operation (Li et al., 2014; Li et al., 2019; Wu et al., 2019; Huang et al., 2020). SDA is an isothermal nucleic acid cyclic amplification technique that displaces the target analyte from the template strand by polymeric growth to cause cyclic amplification of the signal (Qu et al., 2021; Chi et al., 2022; Zhao et al., 2022; Cao et al., 2023). As a hairpin structure DNA self-assembly reaction, CHA can achieve signal amplification by constructing a circular loop of the target strand at room temperature without the assistance of other enzymes (Zhuang et al., 2014; Liu et al., 2019; Xing et al., 2019). Both of these amplification strategies have been extensively used in the field of sensing. A single amplification strategy, however, may still suffer from insufficient sensitivity and limited signal gain. Therefore, the dual signal amplification strategy (SDA-CHA) integrating SDA and CHA is anticipated to provide superior analytical performance. This strategy relies on the extension of telomerase primers into long-stranded DNA in the presence of telomerase to trigger DNA amplification reactions (SDA-CHA) amplifying the detection signal, providing an idea for the detection of enzyme markers. It both ensures the specificity of telomerase recognition and significantly improves the detection sensitivity.

**SCHEME 1:**
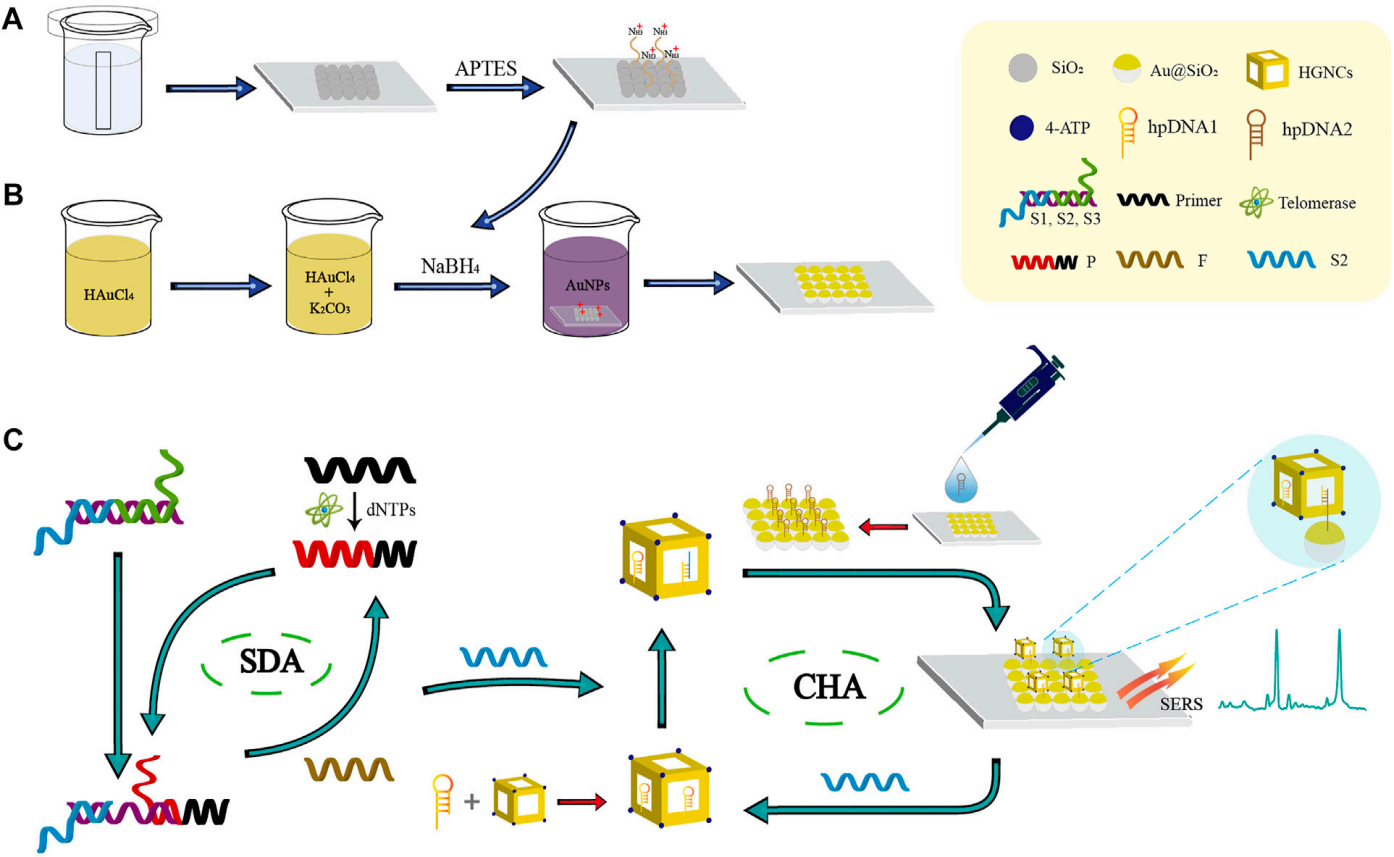
Schematic illustration of the biosensor combined with the SDA-CHA strategy for ultra-sensitive analysis of telomerase activity. **(A)** Synthesis of the single-layer SiO_2_ colloidal crystal film. **(B)** Fabrication of the Au@SiO_2_ array substrate. **(C)** The process of telomerase extension reaction and dual DNA-catalyzed amplification strategy.

In this paper, a novel biosensor with SDA-CHA as the dual signal amplification strategy was constructed for the telomerase detection to realize the early-stage diagnosis of HCC (Scheme 1). The single-layer SiO_2_ colloidal crystal membranes synthesized by vertical evaporation self-assembly was applied for the adsorption of AuNPs *via* electrostatic adsorption to prepare the ordered Au@SiO_2_ array (Schemes 1A, B). The Au@SiO_2_ array modified with hairpin DNA2 (hpDNA2) and Au-AgNCs labeled with Raman molecules and hairpin DNA1 (hpDNA1) were used as the capture substrate and SERS probes, respectively. The presence of telomerase and deoxy-ribonucleoside triphosphates (dNTPs), telomerase primer can form a long-stranded DNA that contains repetitive sequences (TTAGGG) to trigger the SDA reaction and the products can initial the CHA reaction between the SERS probes and capture substrate, attaching Au-AgNCs to the Au@SiO_2_ array’s surface. Thus, the local electromagnetic field (or “hot spots”) can be significantly amplified, leading the enhancement of SERS signal as shown in (Scheme 1C). After the optimization of experimental conditions, the sensing performance including reproducibility, specificity and sensitivity were investigated. Finally, the expression level of telomerase in peripheral blood of patients at different HCC stages were analyzed and ELISA was employed as the golden standard to verify the accuracy. Overall, this novel biosensor has high potential in the early screening of HCC patients.

## 2 Experimental section

### 2.1 Materials

MP-2040 silicon dioxide (SiO_2_), (3-aminopropyl)-triethoxysilane (APTES), potassium carbonate (K_2_CO_3_), sodium borohydride (NaBH_4_), p-aminothiophenol (4-ATP), chloroauric acid (HAuCl_4_), hexamethylenetetramine (HMT), polyvinylpyrrolidone (PVP), silver nitrate (AgNO_3_), ascorbic acid (AA), ethanol, magnesium chloride (MgCl_2_), potassium chloride (KCl), deoxynucleotide solution mixture and telomerase were supplied by DiBo Chemical Technology Ltd. (Shanghai, China). Sangon Inc. (Shanghai, China) designed and synthesized the oligonucleotides used in this experiment, and the sequences were outlined in Table 1. Ethylenebis (oxyethylenenitrilo) tetraacetic acid (EGTA), tris-hydrochloride (Tris-HCl), lymphocyte isolation solution (Ficoll Plus 1.077), phosphate-buffered saline (PBS), and bovine albumin (BSA) were provided by Solarbio (Beijing, China). All ultrapure water was obtained by Milli-Q purifier (resistivity>18 MΩ cm). All beakers were completely cleaned with ultrapure water after being submerged in newly prepared aqua regia, and then dried.

**TABLE 1:** Oligonucleotides applied in the experiment.

Name	Sequences (5'-3')
Primer	AATCCGTCGAGCAGAGTT
S1	ACC​CGA​CGT​ATC​ACT​GAC​TAT​CTG​CCT​AAC​CCT​AAC​CCT
S2	AGT​CAG​TGA​TAC​GTC​GGG​TTA​GGC
S3	GGT​TAG​GCA​GAT​CAT​CGA​C
F	AGG​GTT​AGG​CAG​ATA​GTC​AGT​GAT​ACG​TCG​GGT
hpDNA1	HS-ATACGTCGGGTTAGGCAGATCATCGACGCCTAACCCGACGTATCACTGACT
hpDNA2	HS-AGATCATCGACATACGTCGGGTTAGGCGTCGATGATCTGCCTAACC-NH2

### 2.2 Fabrication of the Au@SiO_2_ array substrate

SiO_2_ colloidal crystal films prepared by vertical evaporation self-assembly method were subjected to amination in a .5 (v/v) ethanol solution of APTES followed by drying after 24 h (Zhang et al., 2011; Deng et al., 2014). AuNPs were prepared by sodium borohydride reduction method. 200 mL of 1 mM HAuCl_4_ was stirred and mixed rapidly, then slowly added 1 mL of .2 M K_2_CO_3_ solution, followed by 9 mL of .5 mg/mL NaBH_4_ solution, with color of the solution turned burgundy after 15 min of stirring. To fabricate the Au@SiO_2_ array, the aminated SiO_2_ colloidal crystal film was placed in the gold nanoparticle solution with constant stirring. Since the aminated SiO_2_ colloidal crystal film was positively charged and the AuNPs were negative, electrostatic adsorption was used to adhere the AuNPs to the surface of SiO_2_ colloidal crystal film.

### 2.3 Preparation of hpDNAs-functionalized Au@SiO_2_ array substrate

The hairpin DNA2 (hpDNA2) was incubated at 95°C for 5 min and then progressively cooled to room temperature. Then 10 μL of activated hpDNA2 was added dropwise to the previously prepared Au@SiO_2_ arrays (by modifying the hpDNA2 onto the surface of ordered Au@SiO_2_ array using Au-S covalent bonds (Li et al., 2016)) and incubated at 25°C for 2 h in a constant temperature incubator.

### 2.4 Synthesis of Au-AgNCs

Au-AgNCs were synthesized by reference to Li’s approach (Li et al., 2018). Firstly, 3 mL of .75 mM HAuCl_4_ was slowly added to 3 mL of .03 M HMT with constant stirring. Then, after the solution was translucent, 3 mL of .30 M PVP and 100 μL of .01 M AgNO_3_ were added to the mixed solution. 50 μL of .08 M AA was added after a brief period of stirring, and the mixture was then agitated for about 20 min as the solution gradually turned dark purple. Next the solution was allowed to stand at room temperature for about 12 h, followed by centrifugation and washing twice with ultrapure water and ethanol, respectively. The prepared Au-AgNCs were dispersed into ultrapure water and kept at 4°C in a dark bottle for the preparation of SERS probes.

### 2.5 Preparation of SERS probes

100 μL of 4-ATP used as Raman reporters (10 mM, in ethanol) and activated hpDNA1 were added to 5 mL of prepared Au-AgNCs (4-ATP and hpDNA1 attached to the surface of Au-AgNCs *via* Au-S covalent bonds) and stirred for 2 h at room temperature. Next, the mixture was centrifuged at 8,000 rpm for 15 min to remove the surplus 4-ATP and hpDNA1 by discarding the supernatant, and the precipitate was dispersed in 80 mL BSA solution (1 wt%). Finally, the mixed solution was incubated (25°C, 2 h) and purified (9,000 rpm, 25 min). Thus, SERS probes (Au-AgNCs@4-ATP@hpDNA1) could be achieved.

### 2.6 Sample collection and processing

Samples of human peripheral blood were obtained from the School of Clinical Medicine of Yangzhou University. HCC is classified into different stages according to clinicopathological stage indicators, which are denoted as I, Ⅱ, Ⅲ and Ⅳ. All experiments were performed in accordance with relevant laws and approved by the Ethics Committee of the Clinical School of Medicine of Yangzhou University. Isolation of peripheral mononuclear cells (PMNC) from peripheral blood samples from HCC patients by Ficoll density gradient centrifugation (720 g, 20 min). The cells were then washed three times in phosphate-buffered saline and precipitated by low-speed centrifugation. Finally, PMNC were lysed, centrifuged at 16,000 g for 20 min, and supernatant was immediately frozen using liquid nitrogen and stored at −70°C.

### 2.7 Telomerase extension reaction

For telomere extension reactions, different concentrations of telomerase solutions (0, 5 × 10^−5^, 1 × 10^−4^, 1 × 10^−3^, 1 × 10^−2^, 5 × 10^−2^, .1, .5 IU/mL) were added to extension solutions containing .5 mM dNTPs and 1 nM telomerase primers (20 mM Tris-HCl (pH 8.3), 1 mM EGTA, 4 mM MgCl_2_, 63 mM KCl and .05% Tween 20). The mixture solution was incubated for 1 h at 37°C and the extension reaction was terminated by thermal denaturation of telomerase (90°C, 10 min). For control experiments, telomerase extracts need to be heat-treated, and the subsequent methods are the same as those mentioned above.

### 2.8 Detection of telomerase activity

Single-stranded (ssDNA) S1, S2 and S3 (100 μL, 5 × 10^−7^ M) were mixed together and incubated for 1 h at 37°C to form complementary double-stranded DNA (dsDNA). Then add telomerase extension product (P), 100 μL of fuel DNA (F, 5 × 10^−7^ M), and 200 μL of labeled probe (Au-AgNCs/4-ATP/hpDNA1). A large number of S2 chain complexes anchored by labeled probes after incubation in a thermostatic incubator at 37°C for 12 h. Subsequently, the probe composite solution was added dropwise to the prepared Au@SiO_2_ array and incubated for another 24 h to allow the probe fully contact the capture array. Finally, SERS measurements were then carried out after the array substrates had been rinsed with ultrapure water to remove any extra SERS probes.

### 2.9 Characterization

The morphology and structure of the nanomaterials were characterized using an S-4800 II field emission scanning electron microscope (Hitachi, Japan) and a Tecnai G2 F30 S-twin field-emission transmission electron microscope (FEI, America). UV-Vis-NIR absorption spectra were recorded with a Cary UV-5000 ultraviolet absorption spectrometer (Agilent, USA). Raman spectra were obtained using a Renishaw inVia micro-Raman spectrometer at 785 nm with a 50× long working distance objective with the laser power and acquisition time set to 5 mW and 5 s, respectively. SERS spectra of 15 different spots on the detection area were randomly selected to obtain the average results to ensure validity and representativeness.

## 3 Results and discussion

### 3.1 Characterization of Au-AgNCs

**FIGURE 1:**
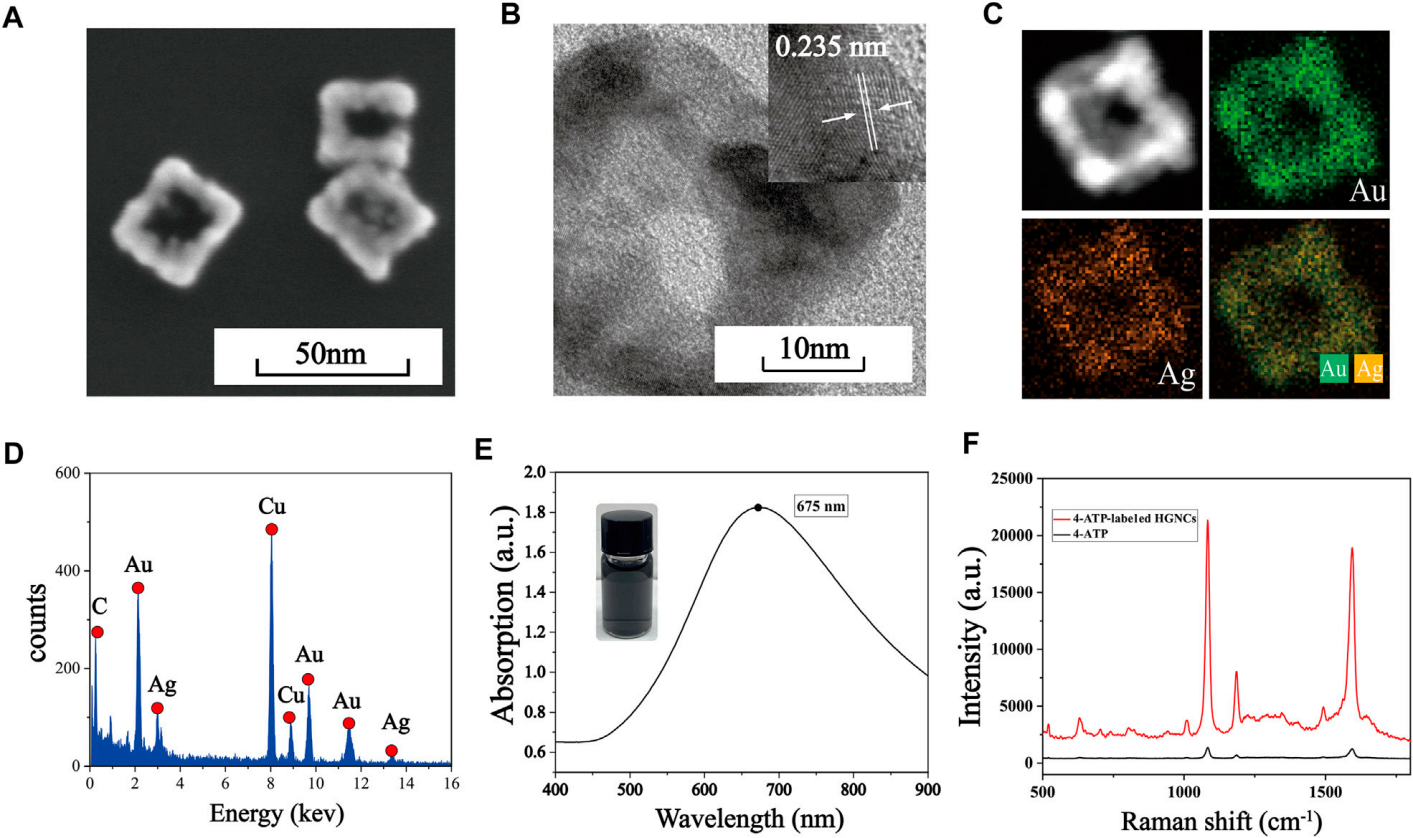
**(A)** SEM image of Au-AgNCs. **(B)** HRTEM image of Au-AgNCs. **(C)** HAADF-STEM image and elemental mappings of Au-AgNCs. **(D)** EDX spectrum of Au-AgNCs. **(E)** UV-Vis-NIR absorption spectrum of Au-AgNCs. **(F)** SERS spectra of pure 4-ATP and 4-ATP-labeled Au-AgNCs.

SEM is used to characterize the morphology of Au-AgNCs, the structure of which are cubic framework with a side length of nearly 30 nm and a wall thickness of nearly 7.5 nm, as shown in Figure 1A. The crystal structure of Au-AgNCs can be observed by HRTEM images (Figure 1B), the lattice spacing of the tip is .235, which corresponds to the (111) plane where Au-AgNCs preferentially grow. To better visualize the structure of Au-AgNCs, the high angle annular dark field scanning transmission electron microscopy (HAADF-STEM) and elemental mapping analysis (Figure 1C) were performed to indicate that Au-AgNCs are mainly composed of gold elements (green) and contain a small amount of silver elements (orange), both uniformly distributed in the hollow structure. After that, the elemental composition of Au-AgNCs was further investigated by energy dispersive spectroscopy (EDS) (Figure 1D), and significant Au and Ag peaks could be found on the spectra, and the peak of Au was higher than that of Ag, indicating that the proportion of Au elements was higher than that of Ag elements. Due to the sample being placed on a Cu grid for detection, the spectrum exhibits high absorption in the 8.0–9.0 keV band, generating a Cu peak. The appearance of smaller C peaks may be due to the conductive adhesive tape which contains elemental carbon. The UV-Vis-NIR absorption spectra of Au-AgNCs are shown in Figure 1E, as can be seen, Au-AgNCs have a strong absorption peak at 675 nm and the inset image indicates that the color of the prepared Au-AgNCs is light black. The SERS spectra of 4-ATP and 4-ATP-labeled Au-AgNCs are shown in Figure 1F, compared to the weak signal of 4-ATP, the 4-ATP-labeled The SERS signals of Au-AgNCs were significantly enhanced, which indicated a significant SERS enhancement effect of Au-AgNCs. 4-ATP had strong SERS signals at 1,090 cm^−1^ and 1,592 cm^−1^, which were caused by the stretching vibration of C-S and C-C bonds (Quynh et al., 2016).

### 3.2 Characterization of Au@SiO_2_ array substrate

**FIGURE 2:**
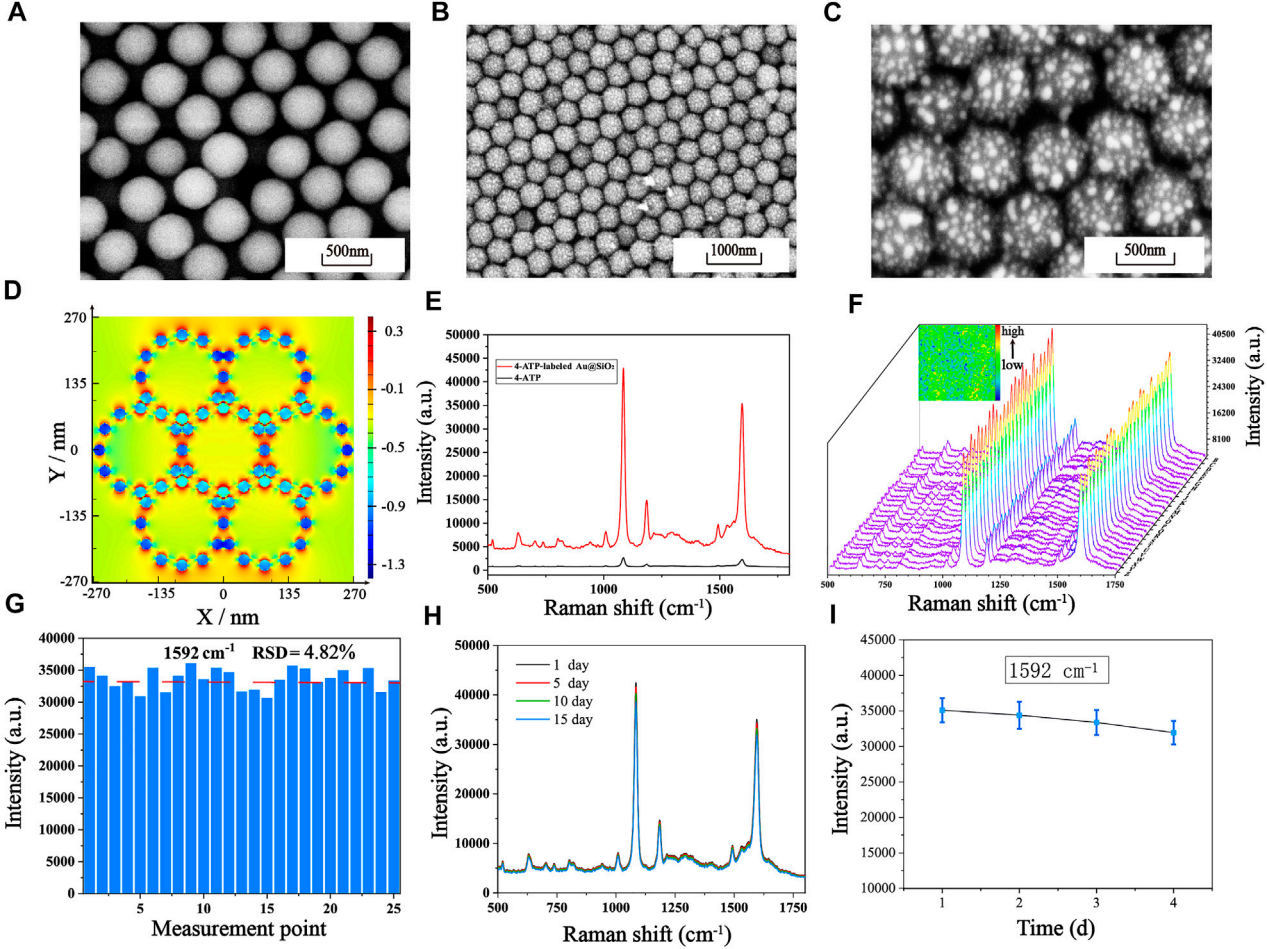
**(A)** Single-layer SiO_2_ colloidal crystal films. SEM images of the Au@SiO_2_ array substrate at different magnifications: **(B)** Low magnification. **(C)** High magnification. **(D)** The simulated electric field intensity distribution images of the array substrate from the upper view **(E)** SERS spectra of pure 4-ATP and 4-ATP-labeled Au@SiO_2_. **(F)** SERS mapping of the 4-ATP-labeled Au@SiO_2_ array substrate and SERS spectra of 25 measurement points on the Au@SiO_2_ array substrate. **(G)** The corresponding SERS intensity distribution of the Raman absorption peak at 1,592 cm^−1^. **(H)** After storage at room temperature for 1 day, 5 days, 10 days and 15 days, the average SERS spectra of the 4-ATP-labeled Au@SiO_2_. **(I)** The corresponding line graph of the signal strength at 1,592 cm^−1^.

The fabrication process of the Au@SiO_2_ substrate has been clearly shown in Schemes 1A, B. The SEM images of the SiO_2_ colloidal crystal film (Figure 2A) demonstrates that the single-layer silica arranged neatly on the hydrophilic silicon wafer with an average diameter of roughly 450 nm. Figures 2B, C show the SEM images of the Au@SiO_2_ array at different magnifications, and the Au nanoparticles (AuNPs) could be observed to adhere orderly to the surface of the SiO_2_ colloidal crystal film and the raised AuNPs can cause significant amplification of the local electromagnetic field (“hot spots”) for signal enhancement. To further investigate the mechanism of SERS enhancement on the Au@SiO_2_ array substrate when exposed to linearly polarized beam irradiation, FDTD simulations were performed. As shown in Figure 2D, the Intensive EM field “hot spots” appear in the “V” shaped gap between the adjacent Au@SiO_2_ microspheres, indicating a significant signal enhancement effect here. In order to further test the SERS enhancement effect of Au@SiO_2_ array substrate, Raman detection of 4-ATP (10^−6^ M) labeled Au@SiO_2_ array and pure 4-ATP (10^−1^ M) was carried out, and the Raman spectra are shown in Figure 2E. The characteristic peaks of 4-ATP are at 1,077 cm^−1^ and 1,592 cm^−1^, and we choose the intensity at 1,592 cm^−1^ to calculate the enhancement factor (AEF) of the Au@SiO_2_ array. According to the equation AEF = (I_SERS_/C_SERS_)/(I_RS_/C_RS_), when C_SERS_ = 10^−6^ M and C_RS_ = 10^−1^ M, the AEF is calculated as 1.35 × 10^6^, indicating that the Au@SiO_2_ array has excellent SERS enhancement effect. The Au@SiO_2_ array has a neat array architecture and outstanding SERS activity, as shown by the characterization and analysis above. The homogeneity and stability of the arrays can also influence the SERS detection results. To investigate the homogeneity of the array, 4-ATP signal molecules (10^−6^ mol/L) were adsorbed on the surface of the Au@SiO_2_ array, and then one area of 50 μm × 50 μm was randomly selected for SERS imaging with the characteristic peak intensity at 1,592 cm^−1^. As shown in the upper left corner of Figure 2F, the color scheme from blue (minimum intensity) to red (maximum intensity) shows the signal intensity of the characteristic peak at 1,592 cm^−1^ at each point, and a more homogeneous green color with only a few blue and orange colors is visible throughout the image, indicating a good homogeneity of the Au@SiO_2_ array. In addition, 25 measurement points on the existing Raman mapping at 10 μm intervals were selected, and the obtained spectra are displayed in Figure 2F, and the bar graph of the Raman intensity at 1,592 cm^−1^ is plotted (Figure 2G). The calculated relative standard deviation (RSD) = 4.82%, which indicates that a small number of voids and stacking in the array has little effect on the uniformity of the array. Finally, the substrate was left at room temperature for 1, 5, 10, and 15 days to investigate the stability of the Au@SiO_2_ array. The waveforms and intensity of the SERS spectra are not significantly different from one another, as shown by the Raman spectra (Figure 2H). Figure 2I further shows that the SERS signal intensity of the Au@SiO_2_ array stored for 15 days only decreased to 91.156% compared to that of the Au@SiO_2_ array stored for 1 day, demonstrating the great stability of the Au@SiO_2_ array. With the above advantages, Au@SiO_2_ array has good prospects for biological immunoassays.

### 3.3 Feasibility of the method for the assay of telomerase activity

**FIGURE 3:**
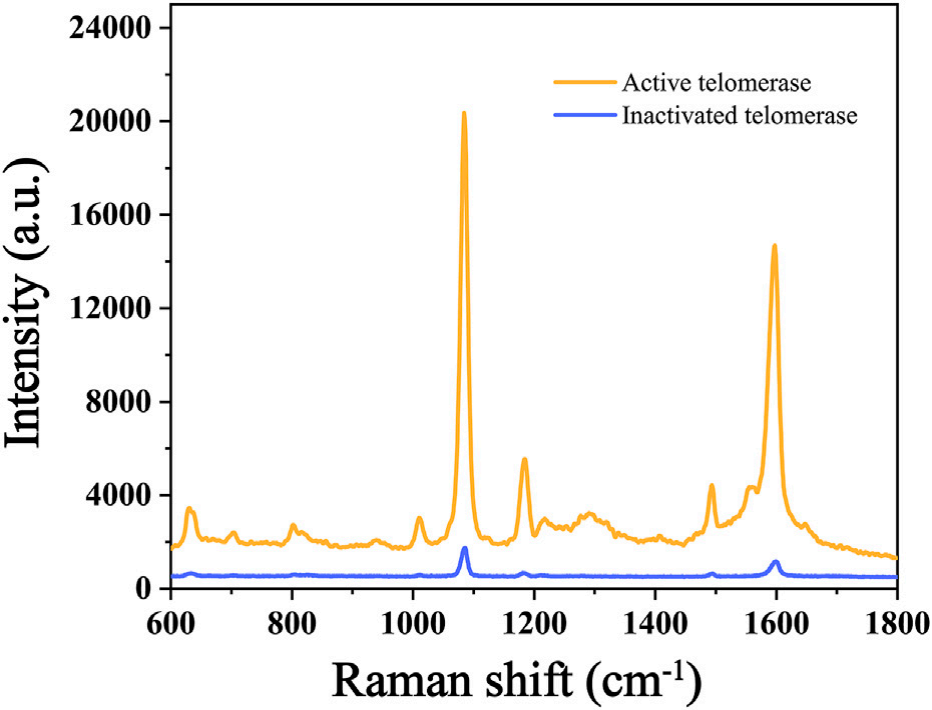
Raman intensity of the control test (active telomerase and inactivated telomerase).

In this experiment, we verified the feasibility of the experiment by comparing the intensity differences of Raman peaks in the conditions with and without telomerase. The concentration of primer used in both experiments was 100 nM, and the DNA concentration, reaction time, temperature and pH values involved in the reactions were the same, excluding other factors that might affect the experimental results. The results of the SERS assay are shown in Figure 3, The Raman signal intensity of 4-ATP in the experimental group with active telomerase was about 20,000 due to the extension of the telomerase primer and the initiation of the subsequent reaction. In contrast, the experimental group with the addition of inactivated telomerase had a Raman signal intensity of only about 1,000 because the telomerase primer could not be extended and the subsequent reaction could not be performed. Therefore, the significant Raman peak signal difference proves the feasibility of the scheme.

### 3.4 Optimization of experimental conditions

**FIGURE 4:**
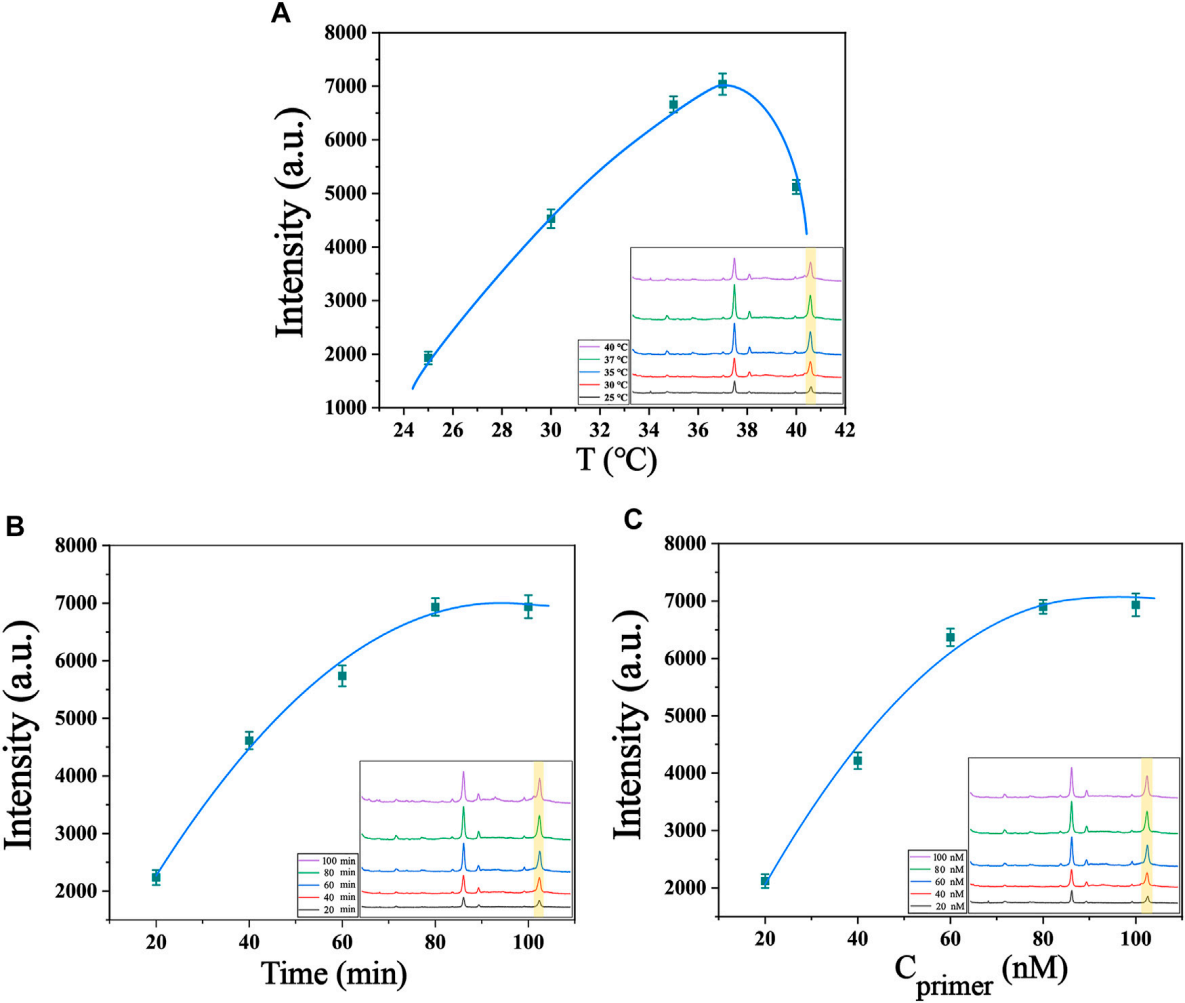
Optimization of experimental conditions **(A)** Optimization of the temperature of the system (telomerase concentration of 1.0 × 10^−2^ IU/mL). **(B)** Optimization of the reaction time of the system (telomerase concentration of 1.0 × 10^−2^ IU/mL). **(C)** Optimization of primer’s concentration of the system (telomerase concentration of 1.0 × 10^−2^ IU/mL). The inset in the lower right corner shows their SERS spectra.

The present biosensor for telomerase detection relies on telomere extension reaction and two cyclic amplification reactions, so optimization of reaction conditions, including system reaction temperature, reaction time and concentration of telomerase precursor, can obtain the best detection efficacy. Temperature has a significant effect on the catalytic activity of enzymes, as enzyme reactions are inhibited at low temperatures, while high temperatures lead to inactivation of enzyme functions. Temperature also affects the collision rate of molecules in the reaction system, so finding the optimal reaction temperature for telomerase reactions is crucial for this experiment. As shown in Figure 4A, the temperature of the system was set between 25°C and 40°C for testing, and it was found that the Raman signal gradually increased and reached the maximum value with the increase of temperature between 25°C and 37°C. Between 37°C and 40°C, the Raman signal gradually decreased with the increase of temperature. Therefore, we chose 37°C as the optimal temperature for the reaction. The reaction time of telomerase also has an effect on the experimental results, because the telomerase activity declines with increasing reaction time. We conducted a series of control experiments, and the telomerase concentrations used in the experiments were all 1.0 × 10^−2^ IU/mL. From Figure 4B, we can see that the Raman intensity changes faster when the reaction time is between 20 min and 80 min, and as the reaction proceeds, the telomerase is depleted, so the Raman intensity changes more slowly from 80 min to 100 min. The Raman signal intensity reached the maximum at 80 min of reaction, so 80 min was chosen as the best reaction time for the system. To investigate the optimum concentration of telomerase primer, we prepared different concentrations of telomerase primer solution for the experiment, as shown in Figure 4C. When the concentration of telomerase primer was from 20 nM to 80 nM, the Raman signal was enhanced with the increase of molecular collision rate in the reaction system as the concentration of primer increased (Roelli et al., 2016). No further significant increase in Raman signal intensity was observed when the concentration was between 80 nM and 100 nM. Therefore, 80 nM is the optimum concentration of telomerase primer. After the optimization of the above experimental conditions, we finally selected the experimental conditions at a reaction temperature of 37°C, a reaction time of 80 min and a telomerase primer concentration of 80 nM for the subsequent experiments.

### 3.5 SERS for the detection of telomerase activity

**FIGURE 5:**
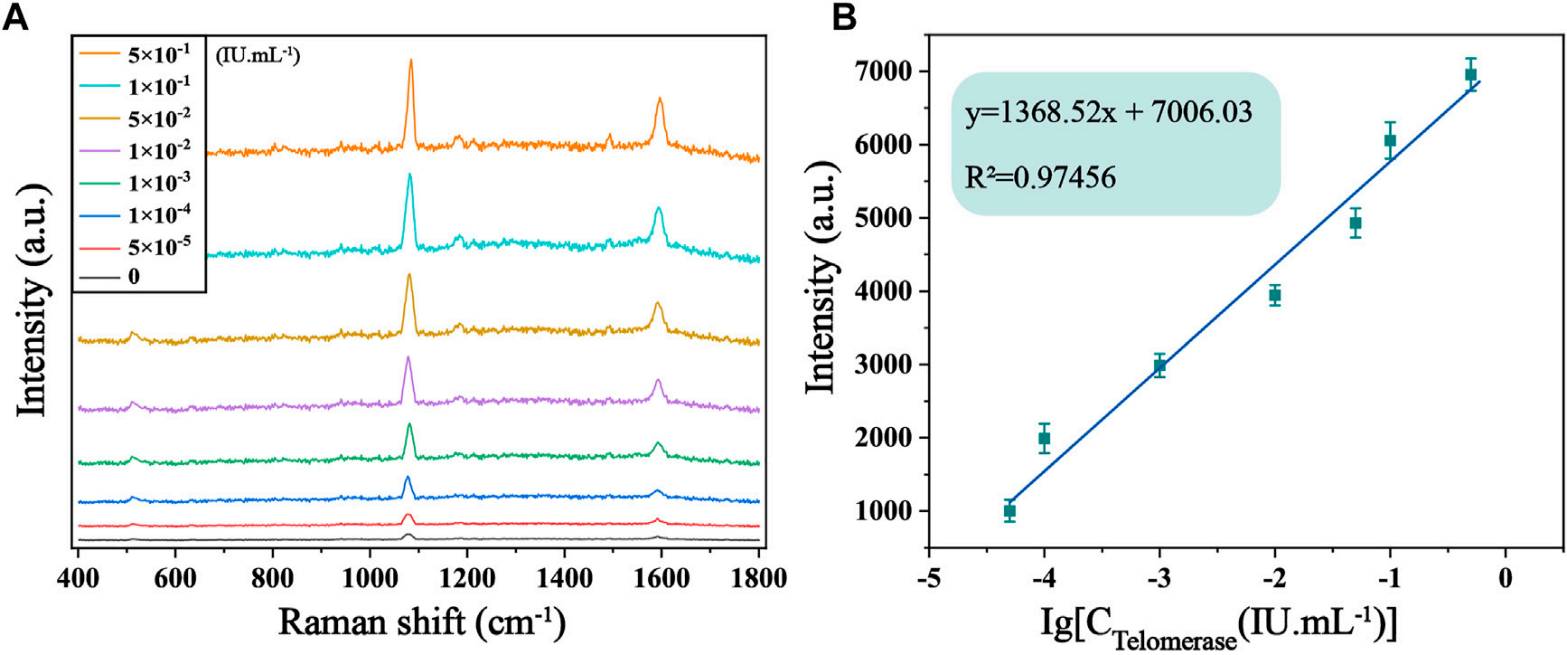
**(A)** SERS spectra of 4-ATP in the presence of telomerase in normal cell lysate solution with different concentrations (0, 5 × 10^−5^, 1 × 10^−4^, 1 × 10^−3^, 1 × 10^−2^, 5 × 10^−2^, .1 and .5 IU/mL). **(B)** Corresponding calibration curves of SERS intensity at 1,592 cm^−1^.

The sensitivity of the biosensor was tested under optimized experimental conditions by adding telomerase to normal cell lysate solution and diluting it to different concentrations, followed by SERS detection. It is evident from Figure 5A that the SERS spectra of 4-ATP in the case of different concentrations of telomerase, and the Raman signal intensity increased with the increase of telomerase concentration. In particular, as shown in Figure 5B, there is an excellent linearity between the signal intensity at 1,592 cm^−1^ and the logarithmic concentration of telomerase. The corresponding linear equation is y = 1,368.52x+7,006.03, the correlation coefficient (R^2^) is .9746, and the limit of detection (LOD) is calculated as 7.59 × 10^−6^ IU/mL, which is lower than other assays. Experimentally, the sensor proved to have considerable sensitivity for telomerase detection. To further assess the feasibility of the method in real sample assays, recovery tests were performed on telomerase samples using the standard processing method described above. The test was divided into two groups, each containing three different concentrations of samples (2 × 10^−3^ IU/mL, 2 × 10^−2^ IU/mL and .2 IU/mL). As shown in Table 2, the recoveries of the six samples were 98.05%, 98.62%, 103.27%, 97.35%, 107.06% and 97.29%, respectively, showing that the biosensor has great feasibility in detecting telomerase in peripheral blood.

**TABLE 2:** Recovery of the biosensor based on the proposed strategy.

Sample group	Added (×10^-3^ IU/mL)	Found (×10^-3^ IU/mL)	Recovery (%)	RSD (%)
Group 1	2	1.961	98.05	5.97
	20	19.724	98.62	5.03
	200	206.545	103.27	6.29
Group 2	2	1.947	97.35	7.21
	20	21.412	107.06	5.63
	200	194.585	97.29	6.56

### 3.6 Specificity of the method

**FIGURE 6:**
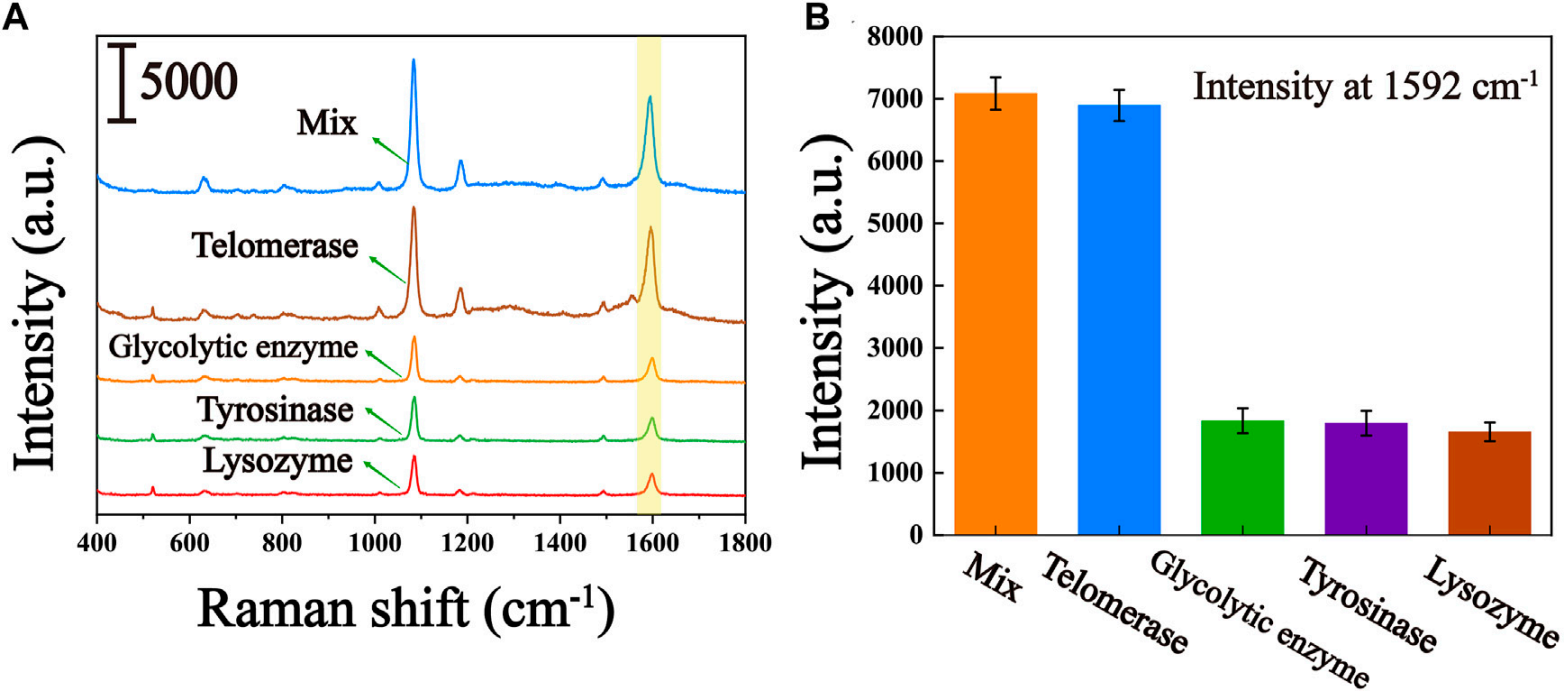
Specificity of the telomerase activity assay (controls include glycolytic enzymes, tyrosine, lysozyme, and their mixtures with telomerase). **(A)** Average SERS spectra of several interferents. **(B)** The corresponding histogram of the peak intensity at 1,592 cm^−1^.

To assess the selective specificity of this biosensor due to the presence of several uncertain interferences in the peripheral blood of the patient, we introduced several interferents in the experiment, including glycolytic enzymes, tyrosinase and lysozyme. As can be seen from the results shown in Figures 6A, B, significantly higher Raman signals were observed for the responses in the presence of telomerase compared to glycolytic enzymes, tyrosinase and lysozyme. Even when mixed with glycolytic enzymes, tyrosinase and lysozyme, the Raman signal was not significantly higher compared to pure telomerase, suggesting that other enzymes present in the peripheral blood of the patient hardly interfere with telomerase analysis. Thus, the proposed biosensor can be used to characteristically detect telomerase activity.

### 3.7 Clinical peripheral blood sample analysis

**FIGURE 7:**
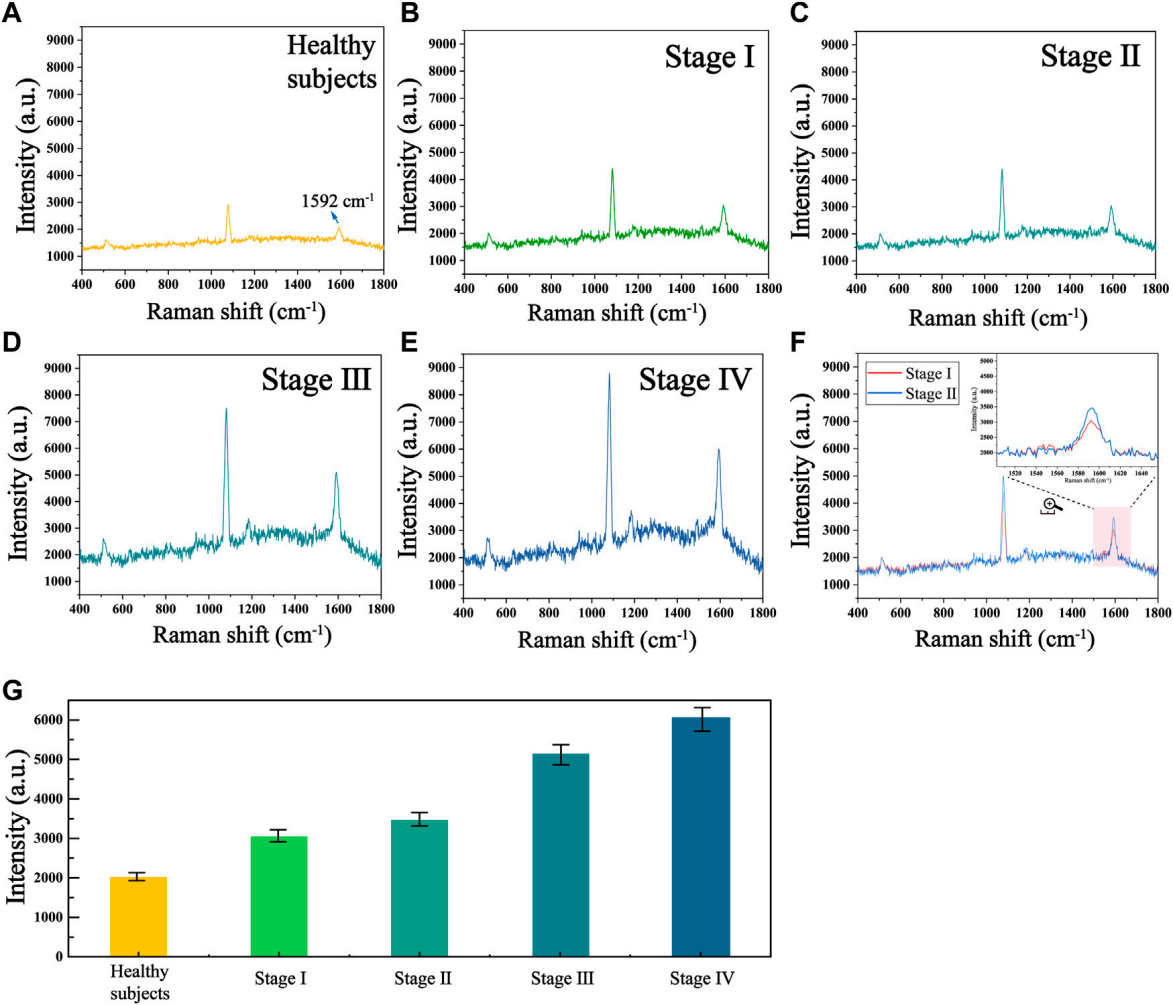
Mean SERS spectra of telomerase in human peripheral mononuclear cells from **(A)** healthy subjects and HCC patients at stages **(B)** I, **(C)** II, **(D)** III and **(E)** Ⅳ. **(F)** Mean SERS spectra at stages I and II. **(G)** The corresponding histogram of the Raman signal intensity at 1,592 cm^−1^.

To further validate the accuracy and reliability of this biosensor in real clinical samples, we performed SERS analysis on 30 healthy individuals and 30 patients with different stages of HCC to obtain the telomerase expression levels in their peripheral blood mononuclear cells. The average SERS spectra of healthy subjects and patients with different stages of HCC are shown in Figures 7A–E. The corresponding bars of signal intensity at 1,592 cm^−1^ are shown in Figure 7F. What can be found is that the telomerase expression levels in the peripheral blood samples of healthy individuals were much lower than those of HCC patients, and the expression levels of telomerase gradually increased as the clinical stage of HCC progressed, which is further evidence that telomerase activity is closely related to the development of hepatocellular carcinoma. Furthermore, by comparing the telomerase expression level detected by ELISA applied as the golden standard, as shown in Table 3, the results detected with this biosensor were not significantly different from those of ELISA, indicating the reliability and accuracy of the proposed biosensor for the detection of telomerase expression levels and its applications in the early screening of HCC.

**TABLE 3:** Mean levels of telomerase activity in clinical blood samples from HCC patients.

Method	Healthy subjects	HCC stage Ⅰ	HCC stage Ⅱ	HCC stage Ⅲ	HCC stage Ⅳ
SERS (×10^-3^ IU/mL)	0.225	1.284	2.603	40.753	183.497
ELISA (×10^-3^ IU/mL)	0.214	1.348	2.498	39.897	185.126
Relative error (%)	5.140	-4.748	4.203	5.905	-4.121

## 4 Conclusion

In summary, we developed a novel SERS biosensor with dual signal amplification based on SDA and CHA for ultrasensitive detection of telomerase. With the assistance of the SDA-CHA strategy, a large number of SERS probes (Au-AgNCs@4-ATP@hpDNA1) were accumulated on the capture substrate (Au@SiO_2_@hpDNA2) to provide numerous “hot spots” for significant Raman signal amplification. Also, when measuring telomerase quantitatively in normal cell lysates, LOD values as low as 7.59 × 10^−6^ IU/mL were obtained well below other approaches previously reported. It is gratifying that biosensor showed excellent sensitivity, specificity and reproducibility for telomerase detection. When used in clinical samples, the method successfully detected telomerase expression levels in the peripheral blood of HCC patients at different stages, whose results matched ELISA well. Overall, this study provides a rapid and ultrasensitive telomerase detection approach, which is beneficial for the early screening of patients with hepatocellular carcinoma.

## Data Availability

The original contributions presented in the study are included in the article/Supplementary Material, further inquiries can be directed to the corresponding authors.
