# Supplementary material for: A dual-amplification strategy-intergated SERS biosensor for ultrasensitive hepatocellular carcinoma-related telomerase activity detection
Source: Front Bioeng Biotechnol. 2023 Jan 13;10:1124441. doi: 10.3389/fbioe.2022.1124441 (PMC9881591; doi:10.3389/fbioe.2022.1124441)
Supplement: Supplementary file 1 [file DataSheet1.docx]

A dual-amplification strategy-intergated SERS biosensor for ultrasensitive hepatocellular carcinoma-related telomerase activity detection

**Kang Shen^1,3†^, Weiwei Hua^3†^, Shengjie Ge^3,4^, Yu Mao^3^, Yuexing Gu^3^, Gaoyang Chen^2^** and Youwei Wang^1^***

^1^ Department of neurosurgery, The Affiliated Hospital of Yangzhou University, Yangzhou, 225000, P. R. China

^2^ Department of Oncology, The second People’s Hospital of Taizhou City, Taizhou, 225300, P. R. China

^3^ Institute of Translational Medicine, Medical College, Yangzhou University, Yangzhou, 225001, P. R. China

^4^ Department of Otorhinolaryngology Head and Neck Surgery, The Affiliated Hospital of Yangzhou University, Yangzhou University, Yangzhou, 225001, P. R. China

* wangyouwei19@126.com

** taizhouchengaoyang@163.com

**†** These two authors contributed to this manuscript equally


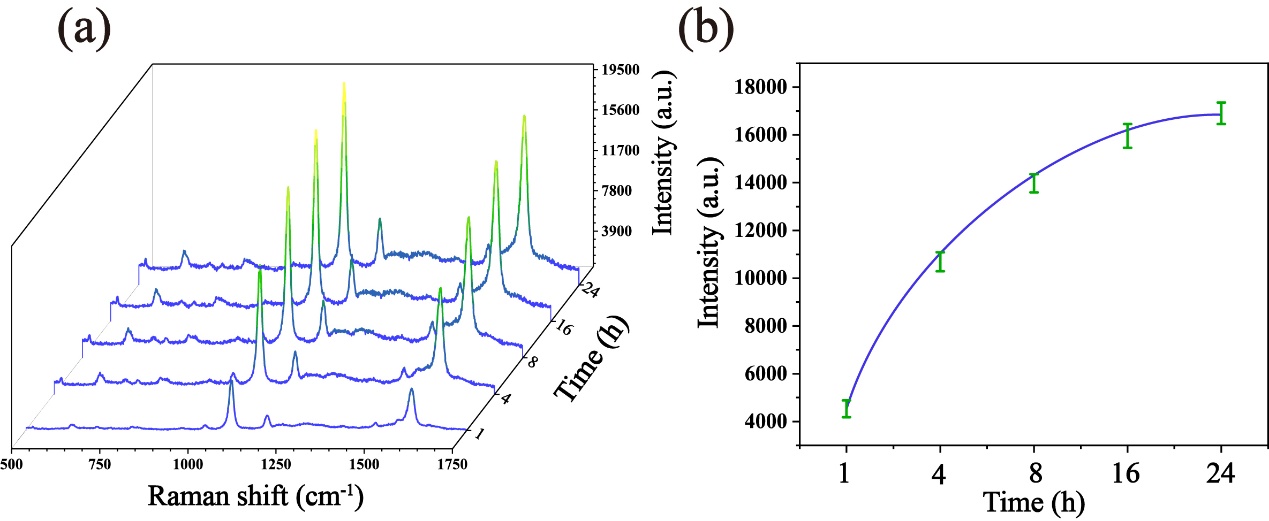


**Fig. S1.** (a) The average SERS spectra obtained after incubation of the probes and Au@SiO_2_ array for different times (1h, 4h, 8h, 16h and 24h). (b) The corresponding bar graph of the signal strength at 1592 cm^-1^.
